# Improving the transition process to independent living for adolescents with profound intellectual disabilities. Experiences of parents and employees

**DOI:** 10.1186/s12913-020-05976-y

**Published:** 2020-12-09

**Authors:** Eirik Roos, Erik Søndenaa

**Affiliations:** 1 Department of Health and Welfare, Municipality of Trondheim, Norway; 2 Faculty of Medicine and Health Sciences (MH), Department of Mental Health, Norwegian University of Science and Technology, 7491 Trondheim, Norway; 3 Department of Brøset, St. Olavs University Hospital, Trondheim, Norway

**Keywords:** Family with intellectual disability (ID) child, Transition process from family home to independent living, Qualitative study

## Abstract

**Background:**

The transition process from the family home to independent living for young adults with profound intellectual disability (PID) becomes delayed. Those families face challenges that exceed those of typical families such as higher objective and subjective burden, more frequent psychological distress and lower social support. The aim of this study was to explore the collaboration process between parents and employees and identify factors that improve the transition with less burden.

**Methods:**

A descriptive qualitative study was undertaken with 18 persons (9 parents and 9 employees) interviewed individually and in groups. In accordance with the municipality’s guidelines, families with a child with PID should apply for housing, when the child turns 16. The purpose is to ensure interdisciplinary collaboration, information flow and coordinated services according to family’s needs. The main question in the interviews was ‘What was your experience with cooperation in the transition process, and what would you do to improve this process?’ The interviews were analysed with a thematic approach using systematic text condensation.

**Results:**

The parents experienced a lack of general information about the ‘housing waiting list’, level of services, and the plan for time of moving from the family home, and how to choose where and whom to live with. Parents described an unsustainable burden of care during the waiting period, and a family crisis caused the allocation of an apartment in a group house. Employees shared challenges to meet families’ wishes, as there were too few group homes. They experienced good collaboration with families and said they offered respite care, due to reduce parents’ burden of care. Employees experienced that PID adolescents developed skills, mastery and degrees of independence after completing a residency at the Folk High School.

**Conclusions:**

To improve the transition process from family home to independent living for young adults with PID, the informants highlighted some factors to reduce the burden of care on families: 1) Systematic follow-up program for families to observe their needs at an early stage; 2) More available group houses; 3) Information about the housing priorities of the services and; 4) Educational preparation programs for families.

## Background

The international prevalence of intellectual disability (ID) in the population is estimated to be about 1% [1]. However, the prevalence as the proportion of the Norwegian population with an ID and significant needs of caring support is 0.44% [2], which corresponds to the term ‘administrative ID’ [3, 4]].

Norway closed all of its institutions for people with ID in 1991, and municipal authorities were called upon to establish locally based services and accommodations; no institutions were left in the country to serve people with an ID. Group homes organised in the municipalities have, for the last 30 years, been the accommodation alternative for almost all adults with ID when moving out of their family home. A strong focus has been put on independence, which further establishes separate housings as the norm for adults with ID [5]. A review of the effect of deinstitutionalisation on the quality of life for adults with ID demonstrated a consistent pattern that moving to the community was associated with improved quality of life (QoL) [6].

Norway, like the other Nordic countries, is characterised by a population of young adults moving away early from their family home. The average age of leaving the family home in Norway was below 20 years in a report from national statistics [7]. In contrast, in the European Union (EU), more than one third (35.3%) of males aged 25 to 34 were still living with their parents in 2017, compared with only one fifth (21.7%) of females in the same age group. On average, around one young adult in four (28.5%) still lives in the parental home [7].

Research on the steps for moving out-of-home for young adults with ID has shown that this move very often becomes delayed and that accommodation out of the home includes many difficulties [8, 9]. There are too few apartments that fit the needs of the person moving out, and inappropriate care and level of services [10, 11]. A large proportion of the parents questioned further chose to delay the established life out of the home to their adult children [12, 13]. Many parents experienced a mismatch between the needs of their child and capacity of the services available, and a needed long-term perspective on the services [9, 14]. However, families with ID children face challenges that exceed those of typical families, and several studies identify the impact of these challenges for the families as higher objective and subjective burden, more frequent psychological distress, and lower social support [15–18].

A shift in the daily responsibility is one aspect of the change for people with ID when they move away from their parents. The parental role is changed from being the primary source of caring to be more supportive. Caring within a family system differs from community caring, and many parents experience these changes as problematic [19–22]. The rights to self-determination of the caring recipient/ child can create conflicts between parents and services. The parental knowledge can limit some of the choices based on previous negative consequences. The parents’ developed competency, life values and expectations concerning their child is not necessarily adopted by the community services. Many parents experience that their child becomes more passive because of their own decision to stay at home instead of participating in social activities. Parents may also describe that they are devalued as resources for their own children, exemplified by not being contacted by the community career’s when their child has significant needs or problems [23–25]. The expectations from the parents may come into conflict with the services. Questions about contact with peers, leisure activities, vacation, health control, daily activities, skills, and relations to other service users often end up in conflicting views. Limitations within the community services, where limited resources are divided between many service users, can make individual adaptations difficult [8, 19, 20, 26].

The relational climate between parents and employees can also be problematic. Values and personality in both parts may end up in likes and dislikes. Employees, who are mandated to work in a team-based ethos with loyalty to their employer (the services), but the parents have their child’s best interest as a primary goal. In addition, many parents have problems with a rule-governed system with no room for spontaneity, while others ask for more explicit care and service planning [8, 12, 22, 24, 27, 28].

This article explores both the parents of children with profound intellectual disabilities and employees’ perspectives in a collaborative process towards making choices for a profound intellectual disability (PID) child about when to move, where, and with whom. The aim of the study is to identify factors that improve the collaboration process, creating less burden.

## Methods

This was a descriptive qualitative study using semi-structured, face-to-face individual interviews, and group interviews. Qualitative methods are well suited for research relating to individual experiences and perceptions [29]. The interviews were conducted between December 2019 and March 2020.

### Ethical considerations

The study was conducted in accordance with the Declaration of Helsinki and was approved by the national Norwegian Centre for Research Data (NSD) in August 2019. The NSD is a Norwegian service provider in CESSDA (Consortium of European Social Science Data Archives), ERIC (European Research Infrastructure Consortium). NSD’s archive is a certified “Trusted Digital Repository”. The participants received written and oral information about the study, and they were informed that they could withdraw at any time. Written consent was obtained before the interviews were conducted, and confidentiality was assured.

### The services to families with a profound intellectual disabilities’ child

The definition of profound intellectual disability (PID) is based on the assessment by the employees at the Health and Welfare Service in the municipality and requires total dependence on personal assistance for everyday tasks when living in a group home. The authors have not validated the information about the families’ children according to checklists and criterion-referenced instruments [30, 31].

In previous literature and studies, we find different designations on how to describe housing for PID children such as clustered housing [32], shared housing [33] and group homes [5]. In our study, we use ‘group homes’ as a term for housing for PID children/adolescents. A group home is a house with 4–10 independent apartments of about 40–50 sqm. In addition, there is a common living area for tenants and a separate office for employees.

The study took place in Trondheim, a city in Central Norway with 200,000 inhabitants. In 2019, the municipality Health and Welfare agency services had registered 436 persons with a diagnosis as ID, aged > 18 and living by themselves; 318 persons lived in group homes with access to staff 24/7 and 118 persons lived in independent housing close to group homes, where they get support from the in-house staff from the group homes.

The municipality has divided the responsibility of Health and Welfare services in two departments; the Child and Family Service (CFS) for inhabitants 0–18, and the Health and Care service (HCS) for inhabitants older than 18. To improve the transition from the CFS to HCS, staffs from HCS join the Patient Care Team (PCT) as the child turns 16. The purpose of PCT is to ensure interdisciplinary collaboration, information flow and coordinated services, where the participants are more committed than if the various service providers are only contacted when needed. In Norway, PCT are not regulated by law or regulation, but are recommended in national guidelines. The PCT also organise the individual care plan, as it is regulated by law. Anyone in need of long-term and coordinated health and care services is entitled to have an individual care plan prepared for them. The plan should only be prepared if the person concerned wants one. The patient’s and user’s goals must form the starting point for the individual care plan. It is therefore important that the patient and user actively participate in the preparation of the plan. Next of kin must also be given the opportunity to be involved insofar as the patient and user permits this. The plan should be continually updated and be a dynamic tool for coordinating and focusing on the services that are provided.

In accordance to the municipality’s guideline, families with PID children should apply for housing to the HCS, as their child turns 16. The HCS must plan housing and services for PID children at 18, and they enroll all the applications on a ‘housing waiting list’ (adults > 18). In September 2019, 20 persons were enrolled on the housing waiting list for group house with access to staff 24/7. The average waiting time (from the age of 18) in 2019 was 25.2 months.

The CFS has various services to support families with ID children such as a Day centre, respite care on weekends in the family home or in temporary municipality group home for adolescents with staff 24/7, and a companion to assist the child to participate in leisure or sports activities in the afternoon. Some of the PID children can be offered a group home for children with in-house staff 24/7. These children must move to a group home for adults when they pass 18. It is always clear at an early stage, that children with profound intellectual disabilities will need group homes with access to staff 24/7 when they turn 18.

There are unequal guidelines to assess needs and assign services to PID children (up to 18) and to PID adults (> 18). The assigned services for children are described in the decision on services as a ‘staffing norm’ as an example; one staff to one child from 07 to 22, 22–07: one in-house staff for eight residents.

The assigned services for adults are described in the decision on services as ‘needs’ in daily activities such as; help with dressing and preparing meals, transport to Day centre, a companion to assist in taking part in one leisure activity for an afternoon once a week. The employees’ main task in the HCS is to assess the needs both parents or dependents and the PID child. The employees are educated as nurses, occupational therapists, social workers, social educators and housing consultants.

### Sample and recruitment

The sample group of the study includes both parents with a PID child aged > 18 and staff in the municipality at the department of Health and Care Services (HCS). The aim was to recruit parents with a PID child aged > 18, where the child has moved out from the family home, or the parents with a PID child aged > 18 living in the family home and the child is on a waiting list for housing. Participants were selected to ensure variation in sex. To recruit eligible parents, the researchers introduced the study to the manager of the HCS both orally and by handing out invitation letter to eligible adults. The manager of the HCS returned with a list of names and phone numbers of adults with a PID child aged > 18, whose child has moved from the family house in 2018–2019, and adults with a PID child aged > 18, whose child is on a waiting list for housing. An overview of the PID adolescents is presented in Table 3.

The researcher (ES) contacted parents from the list by phone and sent an invitation letter by e-mail. Parents were given a choice to be interviewed individually or in a group with other parents, due to personal and emotional experiences that could be difficult to share with unknown people.

All interviews were conducted in a meeting room in a public building, except one individual interview which was conducted in a meeting room at the participant’s workplace.

The aim was to recruit staff with experience in assessing the needs of services to individuals with PID and experiences in cooperating with parents. The manager of the HCS introduced the study only to the staff working with families with PID adolescent being in need for independent living. The manager selected nine employers to participate, with five in one group interview and four in another group interview. The researcher (ER) contacted the employees by phone and sent an invitation letter by e-mail. Both group interviews were conducted in a meeting room in a public building.

### Data collection

Both individual and group interviews were conducted together by the researchers. The interviews were audiotaped and transcribed verbatim. The average time of the individual interviews with parents was 55 min (range approximately 47–75 min), and the average time of the group interviews was 58 min (range approximately 52–68 min). The average time of the group interviews with the staff was 65 min (range approximately 58–72 min).

An interview guide (Additional file 1) was used in all interviews to ensure that all parents and all employees (Additional file 2) were given the opportunity to comment on the same topics. The main questions were ‘Can you tell us about your experience of cooperating with the employees /parents in the transition process from the family house to group house?’ ‘What would you do to improve the transition process from the family home to independent living?’

The follow-up questions addressed what the participants were most and least satisfied with, e.g., the time for moving, option to choose where to move, option to choose whom to live with, and information about the different types of housing, expectations about the waiting list and about the services at the independent housing.

### Analysis

The data were analyses following systematic text condensation, which is a method suited for thematic cross-case analysis inspired by Giorgi’s psychological phenomenology approach [34, 35]. In this study, the analysis process is according to a previous study [36].

First, the analysis started after the first three group interviews with parents were done and continued simultaneously with the recruitment and interview process. The recruitment continued until no new themes emerged from the analysis, and the materials were saturated. Second, the analysis from the interview of employees started after the first group interview was done and continued with recruitment and interview until no themes emerged from the analysis and the materials were saturated.

The analysis itself was also iterative, meaning that the four distinct steps of systematic text condensation were repeated during the whole process. The first step was to read the transcribed interviews with an open mind to obtain a general impression and to identify preliminary themes. Both authors read all interviews and selected, based on richness, two group interviews and one individual interview from parents. In the second step, the transcripts were systematically reviewed line by line to identify meaning units, which were classified and sorted into the preliminary themes. Particularly, at this step, the authors had several meetings to discuss and refine the subthemes and themes. In the third step, the meaning units within each subtheme, established in the second step of analysis, were reduced into a condensate, an artificial quotation maintaining, as far as possible, the original terminology applied by the participants. This facilitated further sorting between the subthemes. In the fourth and last step, the condensates of each subtheme were rewritten in general descriptions, and the final sorting of subthemes into the main themes was finalised.

The whole analysis process was performed and discussed consecutively by both authors. The analysis was validated by a thorough review of the original transcript of each interview to ensure all point of significance was reflected in the results. The quotations that best illustrated the themes were chosen to support the results.

## Results

A total of 18 participants were interviewed (Table 1), nine parents (two men) and nine employees (one man). Of these nine parents were interviewed, seven participated in group interviews and two in individual interviews. Of these nine employees were interviewed (Table 1), all participated in group interview (five in one group and four in one group). All interviews were conducted in a meeting room in a public building, except one individual, in a private workplace. One group interview with employees took place by video, due to the Covid-19 pandemic.

**Table 1:** Characteristics of the informants *(n = 18)*

Number of interviews	Individual		Group		Total
	Sex				
	M	W	M	W	
Parents:					
1			1	1	*2*
1				2	*2*
1				3	*3*
1	1				*1*
1		1			*1*
Employees:					
1				4	*4*
1			1	4	*5*^*a*^
*7 Total*	*1*	*1*	*2*	*14*	*18*

^a^) Video meet

The results are presented under two separate headings due to their informants’ different perspectives. One heading represents result from parents and one result from employees (Tables 1, 2 and 3).

**Table 2:** Characteristics of the employees *(n = 9)*

	Sex		
Employees	W	M	Total
**Number**	8	1	9
**Profession**			
Nurse	1	1	2
Social worker	5	–	5
Housing consultant	2	–	2

**Table 3:** Characteristics of the PID adolescents *(n* = 9*)*

	Sex		
PID adults	W	M	Total
**Number**	2	7	**9**
**Age**			
18–20		2	**2**
20–25	2	3	**5**
> 25		2	**2**
**Living**			
Family home	1	2	**3**
Group home	1	6	**7**

### Results from parents

The findings from the parents were categorised into three themes: 1) Unsustainable burden of care 2) Too large variety in ages and needs of those living in group house 3) Lack of confidence with the services.

#### Unsustainable burden of care

The informants experienced an unsustainable burden of care during the waiting period after applying for housing for the child, especially after they passed 18. The community’s guideline stated that families with an ID child had to apply for an apartment when the child turned 16. All applications were organised in a *‘*housing waiting list*‘*, and most parents expected that their child should move to an apartment at 18.

*‘I do not understand the priority of the waiting list - it is so closed - it is problematic to deal with - the process is not transparent - you do not know anything – I wish they knew what it feels when we are so tired after so many years.’*

As there was a lack of apartments that fitted the needs of their children, they described frustration and exhaustion with the family home situation. The informants told about missing information about where the child should move to or how long they had to stay on a waiting list for an apartment.

*‘I don’t think they (staff in the municipality) understand - and see signs of an exhausted body - they understand it’s tough, but no one realises how tough it really is - I have parents who have helped for many years - but they are also starting to get old – I had to call them one night when I couldn’t do it anymore - there was a crisis - then they came. It hits a whole family.*

Several informants told about insufficient information about services for their child in the family home and an absence of respite care for the parents. Furthermore, several informants said they were not able to work, due to their unsustainable burden of taking care of their child in the family home.

*‘Yes, it is insufficient services from the municipality, even when the children get sick - you must use vacation days or stay home without salary. I must use sickness self-declaration for my own when my daughter is sick.’*

The informants said they were offered an apartment for their child after a family crisis, such as exhausted parents or unexpected illness among them.

*‘We were lucky to receive an apartment in the area close to where we live, as it was unplanned that one of the apartments should be vacant when a family crisis arose.’*

Even after the child has moved into their independent apartment in a group home, most informants experienced an increased burden of care, as they were dissatisfied with the level of services. They felt the need to frequently visit the child to follow up on practical and leisure activities.

*‘It is expected that adults should take care of their children to learn new skills, but when the child turns 18, much is left to them, even though the child still functions on a level as a ‘baby‘. I don’t think it’s okay - they lose their dignity the day they turn 18.’*

One informant said that he worried about the transfer of his child, but in contrast to the others, it was perceived as positive, because the child might receive other stimuli from the staff and other residents, compared to what the exhausted parents could give.

*‘We are definitely worried about relocation, but then he benefits from receiving input from others than exhausted parents after 22 years of care. There is no energy left.’*

#### Too large variety in age and needs living in group house

Most informants experienced too much variation in the age, interests and functional level among residents living in a group house with in-house staff 24/7. It was not planned where they would live, and often they had to accept the allocated apartment, if not, they could be removed from the waiting list. They stated that the most important for the child was to acquire relationships with residents who shared common interests.

*‘I don’t think the municipality is so conscious at allocating apartments based on sex and age. Although large age differences do not mean that they fit together, having common interests are crucial when living together with residents with PID.’*

Furthermore, informants highlighted residents with equal age levels, as one informant experienced that her child at 18 was assigned an apartment among elderly residents. In contrast, an informant expressed satisfaction with the cooperation with the staff in the municipality, as they were connected to well-known children at an equal age level. All had been assigned apartments in a common new building for 10 adults (> 18) some months before the building was erected.

*‘There was a vacancy apartment in new group house - we knew the others who should move in, as my child had grown up with several of them. We could follow the transition process all time and the apartment was adapted to my child’s needs. The group home is superb!’*

#### Lack of confidence with the services

Most informants expressed a lack of confidence with the municipality, due to the lack of individual plans for the time of moving out of the parents’ home and the opportunities to choose an area in the municipality to move.

*‘We have been offered apartments in a completely different area, including apartments without in-house staff. I do not understand why we get such offers, when she is completely dependent on in-house staff.’*

Despite municipality guidelines regarding applying for an apartment when the child was 16, almost all informants acquired an apartment, due to a family crisis. They said that the municipality should rather connect families together, so they could plan housing by themselves.

*‘We want the municipality, through the Health and Care service, to take responsibility to connect [us] with other parents that have children with similar challenges. Then we can join and make an agreement with a property developer to plan a suitable group house with in-house staff. The in-house staff must help to create relationships between the residents.’*

The informants stated that they were concerned about the abrupt reduced service level for their children when the child is 18 years old. When the child lived in a child group house with in-house staff 24/7 until they were 18, they expressed satisfying with the services; it was always one provider to one child. After moving to group house for adults (18+), they told about lack of confidence with the municipality, as the services were reduced by half. They said it was impossible to understand this kind of difference in service level. The functioning level did not improve over the night after entering the age of 18, and the child still was a functional ‘baby’.

*‘He is not 18 years old - he is a baby. He uses pacifier at night because he is not supposed to eat his own clothes. He has toys in his room and likes to be in the sandpit - shouldn’t he be allowed to do so because he’s 18? And that’s not expected? Who needs to decide who he is?’*

They said that employees did not call them to give information about the child’s well-being, nor when they visited their child in the apartment, the employees did not share information.

*‘No, no one from the group house has called me and asked a single question about my son. It is me who must be there and go through his wishes and needs with the staff.’*

### Results from employees

The findings from the informants were categorised into three themes: 1) Too few group house - offer respite care 2) Family crisis elicits acute transition 3) Open and explicit information.

#### Too few group house - offer respite care

The employees told that there were challenges to meet the parents’ wishes and choices for their PID child’s independent housing, as there were too few group homes.

*‘New group house to be built must be approved by politicians and a budget must be allocated to provide services. ’*

The informants said the municipality has a new housing strategy where the purpose is to stimulate more persons with ID to buy their own apartment. However, the informants experienced that most parents want to rent group house and that there must be fellowship with other residents, due to parents being anxious for their children that are placed in an apartment in solitude.

*‘In the choice between a modern independent apartment where people live for themselves or an older group house with accommodation room, I find that many parents prefer group house where they can eat some common meals and have some common activities. Rather a fellowship than an independent housing and loneliness. ’*

The employees said they have a good connection and collaboration with the parents during the waiting transition period to independent housing.

*‘I do not experience being a target – most parents are so well informed about the situation with too few group houses that they know where to direct the frustration. If we have good information and collaboration, then we avoid such frustration.* ’

Furthermore, the informants stated that they offered various respite care to the parents, due to reduce their burden of care.

*‘We try to provide the best possible offer while they wait - what often happens is that they get extended 24/7 residential respite care in weekends, services in the family home, due the great burden of care for the parents.’*

#### Family crisis elicit acute transition

The employees described the waiting period as very exhausting to many parents, after being recommended to apply for an independent apartment when their child was 16, and then being kept uninformed until they suddenly had an apartment offered. The informants said that despite several years standing on the ‘housing waiting list’, it is always families with high risk of crisis that is prioritized when assigned housing.

*‘I can understand that the parents consider the process as unclear. Where is the information? We might send out information every six months on how it is on the waiting list’*

*‘We do not have a queue patch system; we assess the family’s needs and who has the most urgent needs. ’*

The employees pointed on common challenges in the meetings with families with a PID child; expectations of time to move independently, on whom to live with, where to move and what level of services will be offered, fears and thinking of what is best for their child. The informants said they try to provide the best possible offer while they wait. What often happens is that parents get extended respite care, due their unsustainable burden of care.

*‘I think many people get tired of standing in that queue and become willing to accept any alternative after a while. They lower their expectations while waiting. It also happens that one of the parents becomes ill and that the care situation becomes exacerbated. The crises often create emergency placement in respite care 24/7 services in group house. Some people stay there temporarily for a long time, which is not good either.’*

#### Open and explicit information

The informants said they had a good experience of participating in a patient care team (PCT) when the PID child turned 16, as the PCT shared information and is responsible for organising the services that best fit the family. The informants said they were aware that the child’s need for help had been mapped throughout his life and that they were based on this survey when they consider the children’s need for housing and services at the age of 18.

*‘It is important that we participate in a patient care team when the child is 16 years old to inform about what we can offer in terms of housing and services. That's the way it is - parents also need time to adjust and be prepared for the child to move at some point. ’*

Despite, informants telling of young people who had developed skills, mastery and degrees of independence after completing upper secondary education, and later completed a residency at Folk High School (no grades, no rigid curriculum and no exams).

*‘Some of these young people go to Folk High School and have a year away from home. Along the way, we find that the level and need for care is quite different and that they need to manage oneself governs a part.’*

The informants expressed a need for explicit and directly information both through common and individual meetings with parents and children about the children’s development, which opportunities the municipality has to offer of housing solutions, services and school education.

*‘We should arrange common and individual information for parents about alternative forms of housing and a program for moving - we have talked about this for a long time’.*

*‘Expectations must be clarified much more. We have been in close dialogue with parents and the adolescents themselves. What do you see for yourself and what do you think about moving? At the same time, we know that the period from 16 to 20, there is some leap in development regardless of disability or not. A 16-year-old does not intend to sleep for himself, but a 20-year-old may not think of anything else.’*

The informants experienced that some parents want to build private shared housing and ask the informants to connect with other parents with PID children. The informants say that they do not organise such contacts, due to confidentiality.

*‘We are not very good at just that due to handling confidentiality. We sometimes get questions from parents who want to buy or build apartments, so they wonder if we know someone who may be relevant. Unfortunately, we have never got on with it properly.’*

## Discussion

**Fig. 1:**
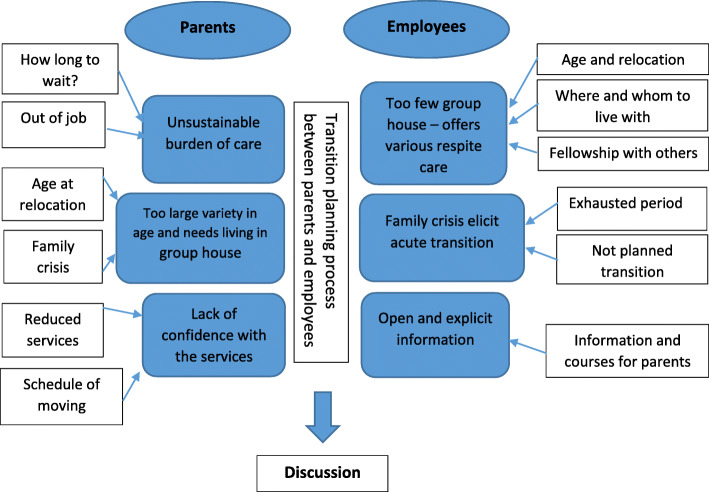
A summarised presentation of the results utilizing the parents’ and the employees’ corresponding themes and subthemes

This study aimed to identify factors that improve the transition collaboration process between parents and employees in the municipality to reduce the family burden. What might be the facilitators of the collaboration with the local municipality? The interviews identified three themes separately, including six subthemes from the parents and the employees (see Fig. 1). The analysis did not result in common themes, but there were common concerns about the process of moving from family home to group home and the level of services. However, the results from parents and employees reveal different expectations of the collaboration on relocation.

### Burden of care – offers respite care

Parent’s preoccupation with the burden of caring, too varied services and mistrust was responded to by the employee’s descriptions of second-best solutions, handling of crises and the need for explicit communication. Importantly, a mutual understanding was more prominent than confronting and conflicting statements.

Although the contrasts were not necessarily very sharp between the two groups of informants, and there was an overlap within the groups, the themes represented dimensions with a potential for conflict. A caring burden described by some of the parents, and their sometimes-unrealistic expectations noticed by the employees, can be a contrary position in this stage of familiar instability. Recent concepts of practitioners’ constructions of families as ‘vulnerable’ have suggested that such a label can be negative on their perceived capacity to parent. Describing families as vulnerable can increase the barriers in accessing appropriate support and a constructive collaboration [37]. The emotional strain caused by experiencing their own child as a burden when negotiating with a professional caring system may cause conflict to both the parents themselves and the collaborating efforts [38]. Although these families felt burdened, local research has documented that such families have a robustness and a family structure similar to typical families, with an even higher proportion of lasting marriages or cohabitation [37].

Several parents stated they were not able to work, due to take care of their adolescents in the family home. In contrast, the employees said they offered various respite care to families during the transition process. These contradictions may indicate an absence of continuous contact between families and employees in the transition process. Thus, it is important to have guidelines with systematic follow-up program for families, to observe their needs at an early stage and to prevent exhausted parents or family crisis [38, 39].

### Too large variety in group house – acute family crisis elicit transition

To face the challenges of too large a variety in group houses, one solution is connecting families with acquaintance adolescents at an equal functional level, age and interest, as an informant experienced. Parents’ worries about social life and social support have also been described in previous studies [9, 26]. The quality of life of the parents and siblings was highly influenced by the wellbeing of their family member who had moved out of the home [26]. The study by Gray et al. [6] found that families experienced seeking housing as stressful and frustrating and that they would like to see social workers and housing professionals acknowledge them as collaborative partners in the process. Similar criticisms were uttered by some parents in the present study; however, these were foremost based on unpredictable circumstances such as health problems or accidents that resulted in crises.

Informants among the employees noticed a positive development in self-care after completing upper secondary education and later Folk High School; placing all PID adolescents resident in a group home at 18 might be counterproductive. The period of moving out from the family home to independent living for young individuals with PID marks a very big change in the life of the person moving out as well as the entire family system, including parents, siblings, friends, neighbours and relatives. The changes and preparations for change when entering adulthood have been studied extensively in the last decade, and a large Australian study (*n* = 340) concluded that most (87%) of parents, but less than two-thirds (59.5%) of young people were involved in general changing processes [20]. What was most interesting in this study was that most collaboration was found regarding indecisions, about education, health services, leisure activities and employment. In relation to accommodation planning, the collaboration between the person, family and the accommodation services was reported only in a minority of the cases (43.9% of the parents and only 21.4% of the persons who were in a situation of moving out).

Most informants (parents) in our study expressed that they were satisfied with the collaboration, according to the assessment of the appropriate level of services, but they were dissatisfied with information about the family’s status on the ‘housing waiting list’.

What characterises an unplanned transition is that the person is offered a random vacant apartment in a group home, regardless of whether the person has the same interests as the other tenants.

Most parents reported that they were listened to first when there was a family crisis. Employees confirmed that a family crisis was one of the most significant reasons for allocating independent living in a group house. Thus, when offered a random vacant apartment in a group home due to family crisis, there is a risk of living with persons having too varied ages, interests or functional levels. To limit the gaps in variation in age, interests and functional levels, one informant said that the employees connected families with acquaintance children at an equal level before the group house was erected. A review [28] including 564 parents and siblings of adults with ID found that adults with ID were more likely to succeed outside the family home when the family engaged in future planning.

### Loss of confidence with services – open and explicit information

The loss of confidence in the community caring services is not a good start of the collaboration of caring for a person with severe needs. Several parents told about a fight with the bureaucracy, ending up with counterparts with a common responsibility including care and dignity. Young people with ID supported by their parents are frequent users of health and caring services. Therefore, an established trust in the system is important to secure the best possible service outcome [40]. When parents in the present study explained how they were placed on a waiting list that was closed to them, with no information about their position in the queue, prospects or progress of this list, it is reasonable that some mistrust occurs.

The employees also commented on the budget control requirements as stressful, and that the economy was a bothersome but needed factor. Employees told about the rigid perceptions that some families had concerning the housing qualities [17], giving a tiny space for negotiating. The main needs reported by the parents in this study was an increased assistance with transition planning, more explicitly related to providing more information about financial assistance, the school transition program and the building of informal community-based support. The worries of parents and sometimes their unfortunate experiences should be attended to when the services are developing in the future. Parents said that they experienced limited opportunities, and the collaboration with the services was both time consuming and stressful [20]. Previous studies have reported that parents experienced the transition process as unsafe and confusing. They lacked knowledge of available services and described the flow of information as deficient [41]. Poor communication between families and care services is described as a critical obstacle to effective transitions to adulthood [42]. Practical assistance so that families can understand the service systems is needed to reduce families’ burdens. A countrywide supervision of municipal health and social services for people with ID (Norwegian Board of Health Supervision, 2017) criticized the municipal services and caregivers for neglecting the relationship between the service recipient and his/her family.

Parents described their mistrust of services when underestimating the needs that the parents had tried to figure out. Some parents worried about the huge diversity in the services and the arbitrariness in the planning phase. They were also upset about the occasional routines of sharing information to the families during the first period after moving. They told that their experiences were not valued, and they were even not asked when caring was planned when the child was moving out. The parents’ concerns about the diminished capacity of their children may have excluded the main person from taking part in the transferring process. The UN Convention on the Rights of Persons with Disabilities emphasizes that transition planning must be done in an accessible way for people with developmental disabilities [43]. This means that information must be adapted with a language, a dissemination and quality assurance so that people with developmental disabilities clearly understand the content. In the present study, parents and community employees were taking decisions on behalf of the main person. Transition plans and program are needed, and the planning should maximise the inclusion of individuals with intellectual disability.

### Strengths and limitations

The study has several strengths. First, the study used common interview guides, both for parents and employees, to detect their perspectives on the cooperation process. In addition, the informants recruited from parents could choose to participate individually or in a group. As two informants chose individual interviews, the study missed important experiences from informants who did not share or discuss their experiences with others. Second, the informants (parents) had various experiences from the process recently, some years ago, and some recently during the cooperation process towards independent living in the group home. Furthermore, some informants had many years of experience in collaboration, as they are still waiting for independent living. Despite this, we achieved a good variation among the informants, but there might have been informants among parents who did not want to be included in the study but could have had other experiences.

Furthermore, an important limitation of the study was that the main persons were not interviewed. By including experiences from the PID adolescents, the study could throw light on their very important perspectives and perhaps calibrate the perspectives from parents and employees. However, persons with profound intellectual disabilities have major challenges in answering questions about when and where to move from family home to independent living according to an informant who said that her 20-year-old child was still a “baby”. The authors’ main reason for not including the main persons in this study was the authors lack of experiences and use of guidelines to ask questions to persons with profound intellectual disabilities. Hence, adolescents with PID were not included, as the aim of the study was to explore the experiences of the parents and the employees.

In addition, the study has not validated the information of the parents’ adolescents, as to whether they meet the criteria to be included as PID.

## Conclusion

To improve the transition process from family home to independent living for young adults with PID, the informants highlighted the following factors that could reduce the burden of care on families:

- Guidelines with a systematic follow-up program for families to observe their needs at an early stage and to prevent exhaustion among parents or a family crisis.
- Avoidance of allocating housing only when there is a crisis; a plan for building more group houses should be presented to local politicians.
- Open information to parents on the ‘housing waiting list’ and improving collaboration after independent living could help parents regain confidence.
- A systematic program should be offered for families with an ID child (for example, on housing options, levels of services, respite care, and age when moving out).

## Supplementary Information

**Additional file 1.** Interview guide for employees.

**Additional file 2.** Interview guide for parents.

## Data Availability

The transcript from the interviews are confidential and will not be shared. A de-identified dataset may be available upon reasonable request of the corresponding author.

## Abbreviations

ID: Intellectual Disabilities
PID: Profound Intellectual Disabilities
QoL: Quality of Life
EU: European Union
NSD: Norwegian Centre for Research Data
CFS: Child and Family Service for inhabitants 0–18
HCS: Health and Care service for inhabitants older than 18
PCT: Patient Care Team
CESSDA: Consortium of European Social Science Data Archives
ERIC: European Research Infrastructure Consortium
UN: United Nations
