# Supplementary material for: Improving the transition process to independent living for adolescents with profound intellectual disabilities. Experiences of parents and employees
Source: BMC Health Serv Res. 2020 Dec 9;20:1133. doi: 10.1186/s12913-020-05976-y (PMC7724626; doi:10.1186/s12913-020-05976-y)
Supplement: Supplementary file 1 — Additional file 1. Interview guide for employees. [file 12913_2020_5976_MOESM1_ESM.doc]

Interview guide for employees
**Theme:**
**The child's age at start-up for leaving home**- when and at what age did you start the process of leaving home?
- was it you yourself who initiated the move or did you get an application from relatives?

**Choice of location and housing**- how do you choose a place and accommodation for the applicant?
- what determines whether the applicant is allocated housing in community or ordinary housing?
- how do you observe the person's need for services?

**Collaboration with relatives**- what experiences do you have in collaboration with relatives about the allocation of municipal housing?
  - were the relatives' wishes for a place of relocation met? and relatives' desire for housing type accommodated?
- was the relative's desire for a place and type of accommodation changed after meeting with the municipality's employees (if employees gave you new information)?
- do you feel that the relatives' recommendations and advice are taken seriously?
- Do you feel that the relatives have a real contribution to the choice of home address and type of residence?
- How is information provided about the accommodation offer? - about the other residents? Are there alternative homes to choose from?
- Do you feel that relatives are involved in measuring the scope of services and the type of services needed for your children?

**Waiting time for housing**- how long has your child been waiting for municipal housing? (number of months)
- do you have ongoing contact with relatives if they have to wait to be allocated housing? How is this contact?
- Do parents and children receive extra help in the parents' home while waiting for housing?

**Offered housing, but thanks no**If relatives have been offered housing, but thanks no;
- what do you think is the reason they refused?

Do relatives have the opportunity to rent / buy ordinary private housing?
- if no, why not?

What can you and your relatives do together to improve collaboration in the relocation process?
