# Supplementary material for: Improving the transition process to independent living for adolescents with profound intellectual disabilities. Experiences of parents and employees
Source: BMC Health Serv Res. 2020 Dec 9;20:1133. doi: 10.1186/s12913-020-05976-y (PMC7724626; doi:10.1186/s12913-020-05976-y)
Supplement: Supplementary file 2 — Additional file 2. Interview guide for parents. [file 12913_2020_5976_MOESM2_ESM.doc]

Interview guide for parents
**Theme:**
**The child's age at start-up for leaving home**
- when and at what age did you start the process of leaving home?
- was it you yourself who took the initiative to leaving home or did you get information and offers from the municipality?

**Choice of location and housing**
- Do you / did you want an area of ​​relocation?
- Do you / did you wish to move to a specific residence? - community or ordinary housing?
- Do you / did you want how much help the child needed after moving out?

**Cooperation with the municipality's employees**
- what experiences do you have in collaboration with the municipality's employees regarding the allocation of municipal housing?
  - were their requests for a place of relocation met? and their desire for housing type accommodated?
- was your desire for location and type of housing changed after meeting with the municipality's employees (if the staff gave you new information)?
- did you find that employees take your advice seriously?
- did you feel involved in choosing a home address and type of residence?
- Did you get to know and information about the other residents, if you were offered a move in community?
- did you feel involved in measuring the scope of services and type of services needed for your children?

**Waiting time for housing**
- how long has your child been waiting for municipal housing? (number of months)
- Do you have ongoing contact with the municipality's employees if there are vacant housing?
- Do you get extra help for your children in the parents' home while awaiting housing?

**Offered housing, but thanks no**
If you have been offered housing, but thanks no;
- why are you saying no?

Do you have the opportunity to rent / buy ordinary private housing?
- if no, why not?

What can you and the municipality do together to improve cooperation in the leaving home process?
